# A Family-Centered Approach to the Care and Rehabilitation of a Child With Sepsis-Associated Encephalopathy

**DOI:** 10.7759/cureus.82671

**Published:** 2025-04-21

**Authors:** Hoang Khanh Chi, Pham Van Minh, Kieu Ngoc Quy, Ngo Hoang Son, Pham Thi To Uyen, Nguyen Van Anh

**Affiliations:** 1 Rehabilitation, Hanoi Medical University, Hanoi, VNM; 2 Pediatrics, Hanoi Rehabilitation Hospital, Hanoi, VNM; 3 Radiology, Hanoi Rehabilitation Hospital, Hanoi, VNM

**Keywords:** family-centered approach, neurological disabilities, rehabilitation, sae, sepsis-associated encephalopathy

## Abstract

Sepsis-associated encephalopathy (SAE) is one of the most common organ dysfunctions, with high mortality rates, lower quality of life, and long-term neurological sequelae. A family-centered approach is the best service delivery method in early intervention and pediatric rehabilitation today. In this paper, we present a 16-month-old child with SAE, resulting in difficulties in multiple developmental areas such as eating, motor skills, cognition, and communication. After leaving the ICU, the child was provided with a care and rehabilitation program including speech therapy, physical therapy, occupational therapy, and oral baclofen for spasticity reduction. With a family-centered approach, the mother was provided information and coached on care and therapeutic exercise routines, and she participated as a member of the treatment team. After two months of rehabilitation treatment, improvements were noted in the child's nutritional status, eating ability, motor skills, cognition, communication, and family satisfaction. This paper emphasizes how a family-centered care and rehabilitation program is implemented in a resource-limited setting.

## Introduction

Sepsis-associated encephalopathy (SAE) often results in a range of long-term challenges, including physical and cognitive impairments, emotional disorders, and communication difficulties [1]. Infants are particularly vulnerable, facing a heightened risk of neurological sequelae, with cerebral palsy affecting approximately 10% of cases [2]. Recognizing the critical importance of early intervention and pediatric rehabilitation, the family-centered approach has emerged as the gold standard for service delivery [3-5]. In the United States, although the family-centered approach has been widely implemented and identified as the best practice in pediatrics, healthcare providers report challenges in translating from theory to practice [6]. In Vietnam, where resources for rehabilitation are limited, such as staff shortages and hospital overcrowding, a family-centered approach in the care and rehabilitation of children with disabilities is being implemented and studied [7, 8]. According to CanChild, a family-centered approach is defined as “a set of values, attitudes, and approaches to services for children with special needs and their families” [9]. At Hanoi Rehabilitation Hospital, we implemented a family-centered model, focusing on three key elements from CanChild's 10 recommendations (#3, #5, and #6). By aligning with the child's condition and the family's aspirations, we formed a multidisciplinary intervention team comprising rehabilitation doctors, nurses, speech and language therapists, physical therapists, occupational therapists, and a nutritionist. This collaborative effort, with the child’s mother as an integral team member, facilitated the child's rehabilitation journey. This paper highlights the benefits and challenges of applying a family-centered approach in a resource-constrained environment for children at risk of neurological disabilities.

## Case presentation

**Figure 1:**
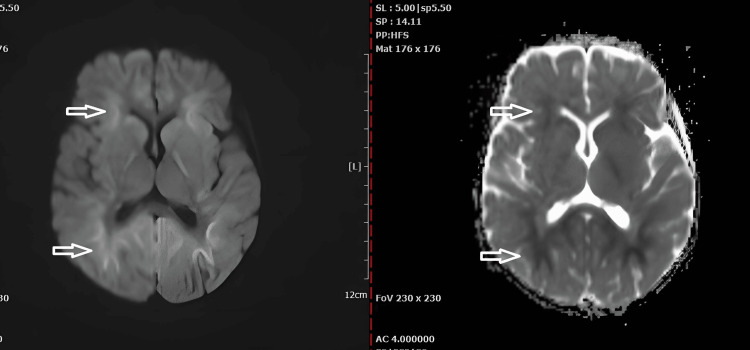
Bilateral symmetrical DWI high and ADC low signal intensity seen at the periventricular white matter (white arrows) shows diffusion restriction. DWI: diffusion-weighted imaging; ADC: apparent diffusion coefficient

A 16-month-old female child, born at full term with a birth weight of 2,800 grams, had previously achieved age-appropriate developmental milestones. However, on December 17, 2024, she presented with vomiting, intermittent high fever, loss of consciousness, and seizures. An MRI revealed lesions in the white matter of both cerebral hemispheres (Figure 1). She was diagnosed with sepsis-induced encephalopathy and received 10 days of intensive care followed by 11 days of medical treatment, stabilizing her vital signs.

Upon transfer to Hanoi Rehabilitation Hospital, the child was awake but unable to differentiate between familiar and unfamiliar individuals, did not produce single words, and exhibited signs of malnutrition with a body weight of 8 kg and choking during feeding. She also presented with spastic quadriplegia, was unable to roll over or control her head and neck, and retained primitive reflexes. Her Hammersmith Infant Neurological Examination (HINE) score was 44, shown to be highly predictive of cerebral palsy.

The rehabilitation doctor conducted an initial assessment and provided the child's mother, who is the primary caregiver, with comprehensive information about the condition and available treatment options. Through collaborative discussions, the intervention team was finalized, including a rehabilitation doctor, nutritionist, nurses, speech-language therapists, physical therapists, and occupational therapists.

Detailed assessments of gross motor skills (Gross Motor Function Measure-66 (GMFM-66)), feeding skills (Dysphagia Disorder Screening (DDS)), informal assessments for communication skills, and fine motor skills were performed, and individualized Goal Attainment Scaling (GAS) goals were established.

The child received 90 minutes of rehabilitation therapy per day in the hospital. Goal-directed therapy (GDT) targeted gross motor functions such as head control, rolling, and sitting for 30 minutes. Individualized speech therapy focused on developing communication skills, such as early communication skills and language comprehension, and expression skills, for 30 minutes. Oral motor exercises for 30 minutes, thickeners, and safe feeding positions were recommended in conjunction with a dietary plan provided by the nutritionist. Oral baclofen was prescribed to reduce muscle spasms during the first month after admission.

To ensure active participation, the child's mother was integrated into the rehabilitation team through training and guidance on care and exercise techniques.

**Figure 2:**
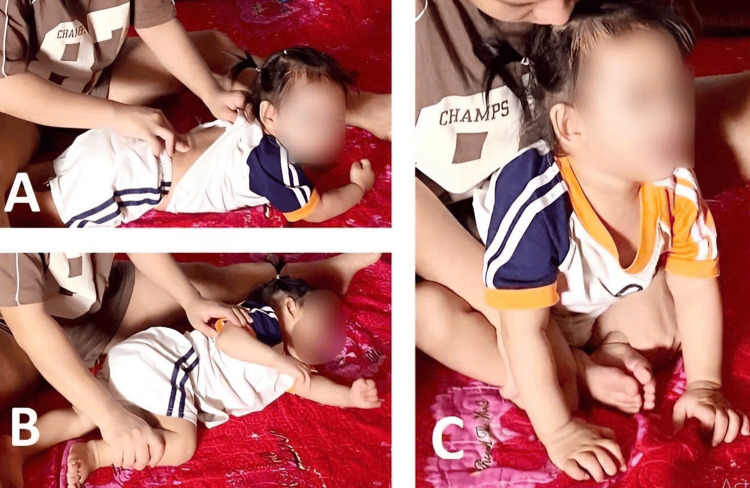
The child's mother practicing gross motor skills with the child A. Head control exercise; B. Facilitation of rolling exercise; C. Facilitation of sitting exercise

This empowered the mother to collaborate in setting and evaluating GAS goals and directly contribute to the child's care and therapy. Every day, the child's mother practiced gross motor skills with the child for 30 minutes under the supervision and guidance of a physiotherapist (Figure 2). Instructional videos from the hospital's YouTube channel were provided to support her efforts. Additionally, handouts and instructions on feeding the child, encouraging communication, and using both hands in play activities were given to the mother, which she reportedly applied to her child.

The intervention team received feedback from the child's mother and promptly addressed her concerns by regular information exchange with her during therapy sessions.

**Figure 3:**
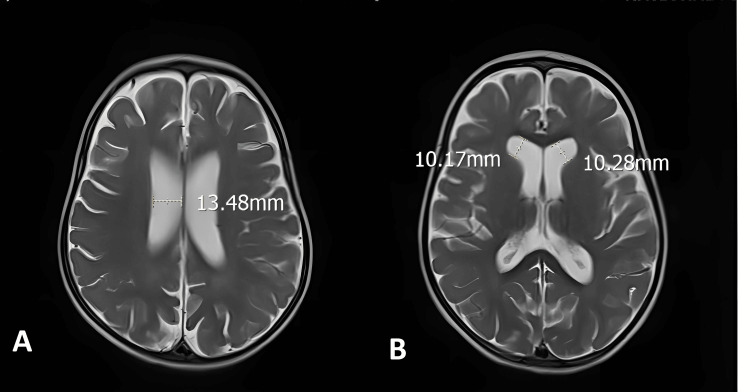
A. Slightly enlarged frontal horns of the lateral ventricles (~10 mm); B. Enlarged bodies of the lateral ventricles (~13 mm)

Following two months of rehabilitation, the child demonstrated substantial improvements in physical and developmental areas. Her attention improved, she understood simple verbal commands, and she verbally produced six single words. Her weight reached 9.6 kg, and her height was 77 cm, both within normal ranges. She no longer choked during eating, and her chewing and swallowing skills were age-appropriate. Muscle spasticity decreased, and sleep improved. She achieved independent rolling and full head and neck control and could sit unsupported for approximately five seconds. Her HINE score increased to 62 (Table 1). The results of the brain MRI on March 19, 2025, showed dilation of the lateral ventricles and the third ventricle (Figure 3).

**Table 1:** HINE scores before functional rehabilitation and after two months of functional rehabilitation HINE: Hammersmith Infant Neurological Examination

HINE	January 7, 2025	March 10, 2025
Cranial nerve function	10	14
Posture items	8	13
Movements	4	5
Tone items	12	17
Reflexes and reactions	10	13
Total score	44 (highly predictive of cerebral palsy)	62 (highly predictive of cerebral palsy)

The mother's evaluation, using the Measure Processes of Care 20-item (MPOC-20), showed that almost all items achieved a score from four to six, and two items showed a score from one to two. Those were as follows: “Let you choose when to receive information and the type of information you want?” and “Provide opportunities for the entire family to obtain information?”

## Discussion

Brain lesions related to sepsis in children are often due to vascular regulatory dysfunction rather than direct damage from infectious agents [10]. An MRI is very useful in excluding other causes of brain dysfunction and is of significant value in predicting SAE prognosis. Several MRI brain lesion findings in the diagnosis of SAE include signal abnormalities in white matter, ischemia, infarction or thrombosis, brain volume reduction, and diffuse cerebral edema. The presence and severity of MRI abnormalities related to sepsis can predict the risk of mortality, neurological disability, and length of hospital stay. The HINE score, a neurological assessment scale for children under two years old, is significant in diagnosing, predicting, and monitoring sequelae of brain lesions, especially cerebral palsy, in this age group. In children under two years old with risk factors, MRI and the HINE score are useful tools for early diagnosis and predicting cerebral palsy, with sensitivity and specificity exceeding 90% [11].

A family-centered approach not only helps children with disabilities achieve their goals and skills, with faster developmental progress, but also provides parents with family-centered care, leading to better mental health, reduced stress, and depression [12]. Simultaneously, studies indicate that the feasibility and effectiveness of services can be affected by various factors. Therefore, when implementing family-centered services, it is necessary to base them on the actual situation to identify priority requirements, evaluate implementation effectiveness, and make appropriate modifications to enhance the family-centered nature of the service [13,14,15].

Based on CanChild’s 10 recommendations for creating family-centered services and actual resources, we focused on three main recommendations: #3, #5, and #6, which are as follows: Promoting collaboration in goal setting, providing information to the family, receiving feedback from families, and addressing their concerns. Additionally, other recommendations are being implemented gradually. However, three out of 10 CanChild recommendations (#1, #7, and #10) have not been implemented due to staff shortage and lack of workspace. These are also the challenges that some hospitals face when implementing family-centered approaches. The issues noted include the provision of information to all family members and delivering it in the manner and timing that the family prefers. An overburdened work environment in hospitals with rigid visiting schedules, which lack flexibility to meet families' needs, is one of the contributing factors, as also highlighted in previous literature [13,14].

In this case, we used the MPOC-20, a self-reporting measure of parents’ perception of the extent to which the health services they and their child(ren) receive are family-centered. Each item is answered on a Likert scale from one to seven, with one representing “not at all” and seven representing “to a very great extent.” The mother's evaluation indicated high satisfaction with the comprehensive coordination and collaboration between professionals and herself. The mother felt supported and respected, and information was consistently shared. However, two areas were identified for improvement: “Let you choose when to receive information and the type of information you want?” and “Provide opportunities for the entire family to obtain information?” [6,9]. We discussed some solutions to support the entire family of the patient, such as increasing the number of available tutorials on the hospital's YouTube channel and developing a telerehabilitation program.

Thus, when developing and implementing a family-centered approach, we identified three priority objectives for implementation. Although three recommendations have not been implemented, other recommendations are gradually being improved, helping the hospital's services become more family-centered over time.

## Conclusions

In the case of a child with SAE, a family-centered approach was implemented in the care and rehabilitation program, guided by the child's condition, MRI findings, and HINE scores for prognosis. This approach initially demonstrated success, evidenced by the child's progress and the family's satisfaction. By prioritizing CanChild recommendations #3, #5, and #6, the family-centered methodology proved effective in supporting the child at risk of neurological disabilities. Addressing challenges such as staff shortage and hospital overcrowding remains vital to further enhancing the family-centered nature of hospital services over time. Some solutions to support the entire family of the patient have been suggested, such as increasing the number of available tutorials on the hospital's YouTube channel and developing a telerehabilitation program.
